# Single-cell metagenomics: challenges and applications

**DOI:** 10.1007/s13238-018-0544-5

**Published:** 2018-04-25

**Authors:** Yuan Xu, Fangqing Zhao

**Affiliations:** 1 Computational Genomics Lab, Beijing Institutes of Life Science, Chinese Academy of Sciences, Beijing, 100101 China; 2 University of Chinese Academy of Sciences, Beijing, 100049 China

**Keywords:** metagenomics, bioinformatics, single-cell genomics

## Abstract

With the development of high throughput sequencing and single-cell genomics technologies, many uncultured bacterial communities have been dissected by combining these two techniques. Especially, by simultaneously leveraging of single-cell genomics and metagenomics, researchers can greatly improve the efficiency and accuracy of obtaining whole genome information from complex microbial communities, which not only allow us to identify microbes but also link function to species, identify subspecies variations, study host-virus interactions and etc. Here, we review recent developments and the challenges need to be addressed in single-cell metagenomics, including potential contamination, uneven sequence coverage, sequence chimera, genome assembly and annotation. With the development of sequencing and computational methods, single-cell metagenomics will undoubtedly broaden its application in various microbiome studies.

## Introduction

Recently, with the development of sequencing technologies and the progress of bioinformatics, high throughput sequencing has been broadly applied to study the composition, function, evolution and interaction of microorganisms in various environments. It directly prompts the blossom of microbial ecology and produces a lot of useful and applicable scientific achievements, particularly in the area of the gut microbiota and human health.

The applications of sequencing technologies on environmental microbiology can be mainly divided into target sequencing, metagenomic sequencing and single-cell genomic sequencing, according to the problems they need to be solved. Target sequencing also called amplicon sequencing, which sequence specific marker genes of microbes such as 16S ribosomal RNA (16S rRNA), ITS, ammonia monooxygenase subunit A gene, or methyl-coenzyme M reductase alpha subunit gene, etc. The 16S rRNA gene is the most popular marker gene for target sequencing, which can solve one important question related to microbial ecology as “who is there” by assigning the reads to a taxonomic lineage based on known 16S rRNA database such as green genes (DeSantis et al., 2006), SILVA (Quast et al., 2013) or RDP (Cole et al., 2014). However, the 16S rRNA reads do not contain enough resolution in identifying bacteria at the species or strain level. In addition, the functions of these microbes cannot be directly determined. Metagenomic sequencing is also called environmental genomic sequencing or community genomic sequencing, which sequence the whole genome of all microbes in the environment. This method can help answer two important questions related to microbial ecology as “who is there and what are they doing” by annotating the reads to known functional gene database such as NR, KEGG (Kanehisa et al., 2008), eggNOG (Huerta-Cepas et al., 2016), etc. The main advantage of this approach is that it provides a comprehensive understanding of the community structure at a high resolution and potential metabolism pathway associated with microbial community (Liu et al., 2013). However, difficulties in metagenome assembly and functional annotation are bottlenecks of metagenomic sequencing, which cannot give a consensus microbial composition compared with 16S rRNA profiles. To overcome this problem, Zhang et al. recently proposed a method RiboFR-Seq (Zhang et al., 2016) for capturing both 16S rRNA variable regions and their flanking protein-coding genes simultaneously, which can help link metagenomic contigs to their 16S rRNA profiles. Spencer et al. introduced a new technique named epicPCR (emulsion, paired isolation and concatenation PCR) to link functional genes and phylogenetic markers in uncultured single cells (Spencer et al., 2015). However, both approaches only partially solve the problem and cannot link all the functional genes of one microbe to its phylogeny.

Due to the weaknesses of target sequencing and metagenomic sequencing, single-cell sequencing is becoming a powerful complementary approach, which aims at sequencing target bacteria at single cell levels. The first step of single-cell genomic sequencing is to isolate single cells from environmental samples using serial dilution, microfluidics, flow cytometry or micromanipulation. The following steps involve in DNA extraction, phylogenetic identification by 16S rRNA gene PCR, multiple displacement amplification (MDA), library construction, sequencing and data analysis. The major advantage of this method is that it can easily link metabolic functions to specific species. In addition, this method can generate a high-quality genome for species with low abundance, which may be lost by using the metagenomic sequencing method. Using the assembled genomes, researchers can investigate genome rearrangement, gene insertion, duplication, gene loss, intra-species variation and virus-host interaction of uncultured microbes. The weaknesses of this method are as follows. Firstly, the cell sorting procedure is complicate and time consuming. Secondly, the highly uneven read coverage and an increased proportion of chimeric reads can be caused by the MDA procedure. Finally, contaminated bacteria or DNA may fail the total experiment.

**Figure 1:**
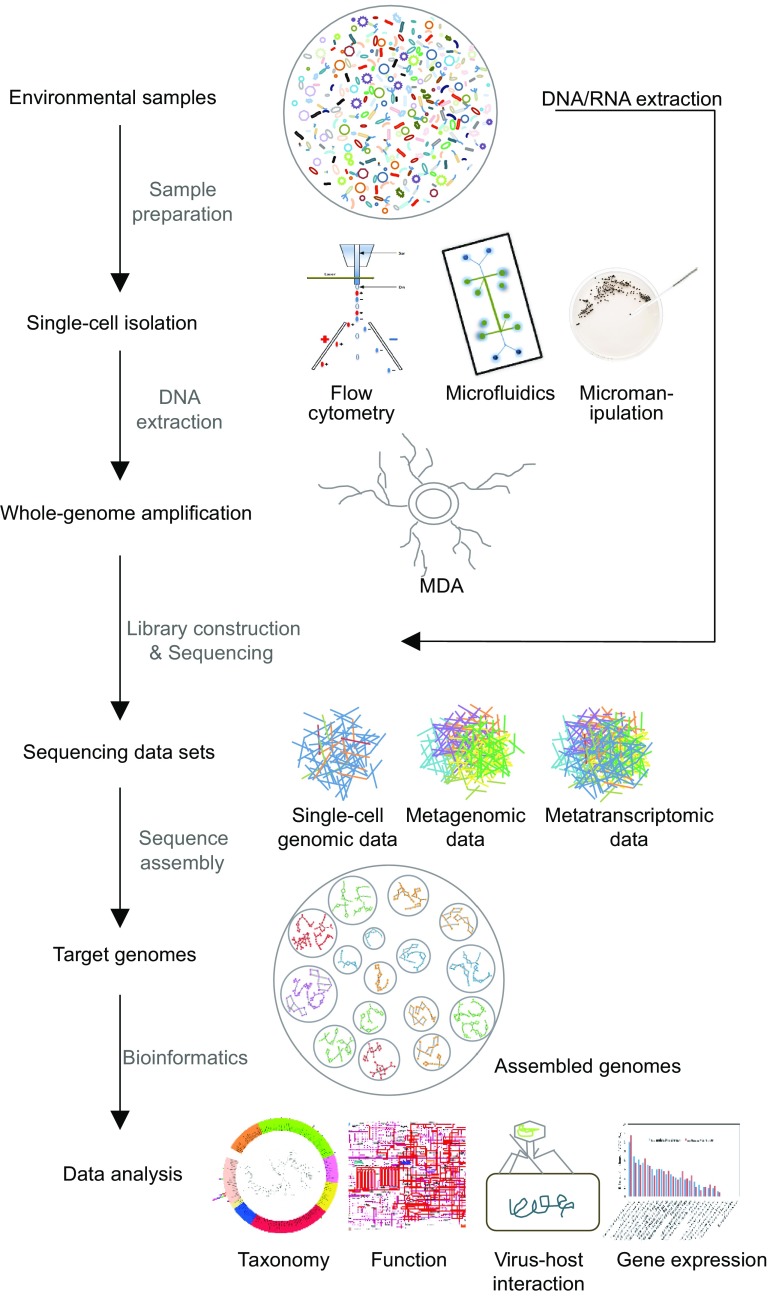
Workflow of single-cell metagenomics

As shown above, these sequencing technologies have their own advantages and disadvantages and they can complement each other in practical applications. For example, metagenomic sequencing is not bothered by problems such as cell-sorting, chimeric reads and uneven read coverage associated with single-cell genomics. Meanwhile, single-cell genomics can offer direct links of species and their functions, which is an important problem that metagenomic sequencing needs to resolve. The combination of these two technologies can greatly solve the challenges faced by each of them. For example, single-cell genomics can provide phylogeny, nucleotide frequency composition and gene content information for metagenomic data binning. Conversely, metagenomic reads and contigs can significantly improve single-cell genome assembly (Blainey, 2013; Dodsworth et al., 2013; Becraft et al., 2015; Ji et al., 2017). Here we will review and discuss the experimental and analytical workflow (Fig. 1) and potential challenges related to the combination of single-cell genomics and metagenomics.

## Whole genome amplification using MDA

**Figure 2:**
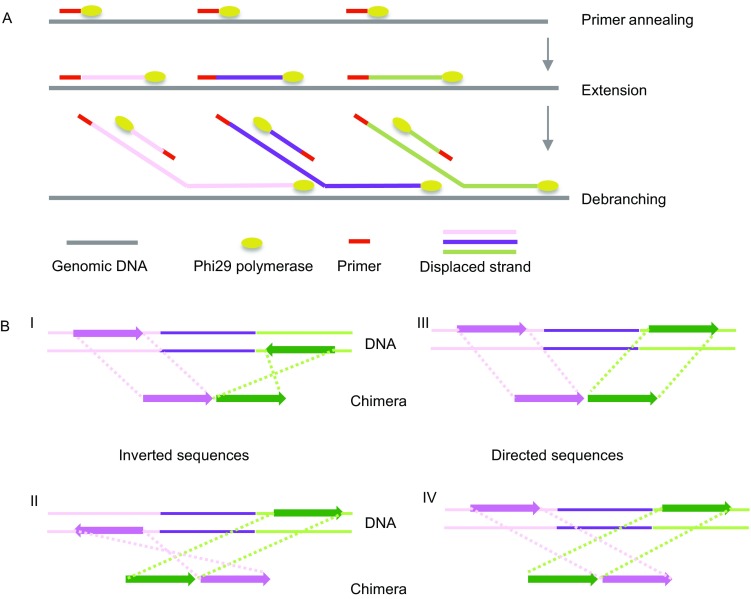
Multiple displacement amplification process and chimera types. (A) Primer Phi29 DNA polymerase annealing to the DNA and extension by Phi29 DNA polymerase. Phi29 DNA polymerase can displace downstream 5′-termini DNA strand to extend the growing 3′-termini strand by a simple branch migration reaction, and 3′-termini can be displaced as well in a similar way. (B) Four types of chimeric rearrangements. I: the second segment is inverted from its original orientation and directly joined after the first segment. II: the second segment is inverted from its original orientation and directly joined before the first segment. III: two directed segments are directly joined. IV: two directed segments are reversely joined

Considering that a bacterial cell generally contains femtograms of DNA and the minimum demand for high throughput sequencing is micrograms, whole genome amplification is an essential step for single-cell genomics. MDA is widely used for single-cell whole genome amplification, which uses random hexamer primers and Phi29 DNA polymerase for large DNA fragment production under isothermal conditions (Fig. 2A) (Blanco et al., 1989; Yilmaz and Singh, 2012). Phi29 DNA polymerase can displace downstream 5′-termini DNA strand to extend the growing 3′-termini strand (Chen et al., 2014) by a simple branch migration reaction, and 3′-termini can be displaced as well in a similar way. However, this method has its own limitations such as false amplification of contaminates, formation of chimeric reads (Lasken and Stockwell, 2007) and production of uneven read coverage. All of these caveats, however, can be largely resolved by downstream computational analyses. Most recently, a novel MDA method (Stepanauskas et al., 2017), WGA-X, used a thermo-stable mutant phi29 polymerase to recover a greater proportion of single-cell genomes, providing another promising strategy to improve single-cell genome recovery.

## Removal of contaminated DNA and sequences

DNA contamination is one of the major challenges needed to overcome before MDA, as the MDA procedure may magnify the contamination and finally lead to the failure of the experiment. Generally, contaminations come from three sources (Yilmaz and Singh, 2012; Blainey, 2013), the tainted specimen during the cell-sorting step, the polluted reagents or equipment used for the experiment, and the inappropriate environment during the experimental process (Blainey, 2013). There are three kinds of solutions and the combination of them can greatly eliminate such contaminations. The first solution is to use strict cleaning measures to guarantee the process of experiment, including ethylene oxide treatment of laboratory disposables (Shaw et al., 2008), heat-sensitive DNA nucleases (Champlot et al., 2010) or UV irradiation (Woyke et al., 2011) treatment of reagents or HEPA-filtered environment (Swan et al., 2011). The second solution is to reduce the reaction volume, which will increase the ratio of single-cell microbial DNA to contaminated DNA because it lowers the reagent-based contamination (Rinke et al., 2014). In addition, a negative control in the experiment is necessary. The last solution is to use computational approaches to identify and remove contaminated DNA after sequencing. For example, contaminated DNA can be identified and removed by aligning all reads against the reference genomes such as human or all currently available microbial genome sequences. However, if the contaminated genome is similar to the target genome, this strategy has the risk to lose sequences aligned to their conserved regions. In addition, tetramer frequency-based composition analysis can be used to remove contaminated sequences (Woyke et al., 2009). Its drawback is that it is computationally expensive, while doing this step after genome assembly can reduce the computational cost.

## Uneven genome coverage

Uneven genome coverage is another key issue in single-cell genomics, which is caused by stochastic primer binding and preferential amplification of some genomic regions during the MDA step (Dean et al., 2001; Hosono et al., 2003; Raghunathan et al., 2005; Zhang et al., 2006). There are two possible strategies to overcome uneven genome coverage. One strategy is to optimize the experiment process such as reducing reaction volumes to increase effective template concentration for MDA (Marcy et al., 2007) or combining the DNA samples of the same species for MDA (Raghunathan et al., 2005; Kvist et al., 2007) or using duplex-specific nuclease to degrade high abundant sequences after MDA on the bases of their re-annealing kinetics (Yilmaz and Singh, 2012). This strategy not only improves the evenness of read coverage but also can increase the coverage of the target genome (Rodrigue et al., 2009). Another strategy, which is more common and often used before metagenomic assembly, is to normalize the sequencing reads using bioinformatic methods (Rodrigue et al., 2009) such as screening and trimming the reads according to their k-mer depth. Reads with high-abundance or unique k-mers can be removed or trimmed before performing sequence assembly (Swan et al., 2011). Some assembly softwares for single-cell genomics have embedded this step in their algorithms such as SPAdes (Bankevich et al., 2012), EULER+Velvet-SC (Chitsaz et al., 2011) and IDBA-UD (Peng et al., 2012).

## Chimeric fragments caused by MDA

Sequence chimera is another serious problem caused by MDA, where different regions of the same or different genomes may be falsely amplified into one fragment during the process of amplification. Lasken and Stockwell summarized different types of chimeras in a single-cell sequencing study of *E*. *coli* and speculated their formation mechanisms (Lasken and Stockwell, 2007). According to their results, chimeras in MDA can be divided into four types as shown in Fig. 2B. The first two types of chimeras showed up when the second segment was inverted from its original orientation along the reference genome, accounting for 85% of the total number of chimeras. The last two types were joining of two segments in a direct orientation, accounting for 15% of the total chimeras. The order of the two segments could also be reversed during the DNA rearrangement. That is, the first segment in the chimera could be joined to a segment that was either downstream or upstream in the genomic sequence (Fig. 2B). Once chimeras are generated and sequenced, which will cause DNA rearrangement and complicate downstream genome assembly. Thus, they need to be identified and removed from the dataset using bioinformatic tools before genome assembly. Reference based chimera check is the main strategy to eliminate such chimeras. Considering that some single-cell sequenced microorganisms may lack reference genomes, the combination of single-cell genomics and metagenomics can make up for this deficiency, as the assembled contigs can serve as the reference to correct chimeras. Recently, Marshall used an iterative “jackknife” procedure by Newbler to exclude chimeric sequences generated by the MDA process without the aid of reference genomes (Marshall et al., 2012). Several algorithms and tools such as UCHIME (Edgar et al., 2011) and DECIPHER (Wright et al., 2012) use sequence frequency information to detect chimeras and are efficient in identifying chimeric sequences in amplicon sequencing, which assume that chimeric sequences are less frequently represented in a given dataset than normally amplified genome regions.

## Assembly of single-cell genome

In the past few years, the accumulation of microbial genomic data increased rapidly. However, the amount of complete microbial genomes increased much slower. The main reason is due to the fact that short sequencing reads, high complexity and unevenness of the environmental samples are limiting factors in metagenome assembly. Assembly is the process of merging overlapped short reads into longer contiguous sequences. Current assembly algorithms are mainly based on read overlap or de Bruijn graph approach, and the combination of these two strategies dominates high-throughput sequencing genome projects (Shi et al., 2017).

It is difficult to get the complete microbial genome from environmental samples with a high diversity of microbes. Generally, researchers only get a pile of gene-centric data, which is difficult to conclude which genes are clustered together in a single organism. Sometimes, when the sample is simple such as extreme environmental samples or enriched samples, researchers may get more complete genome of the most abundant microorganism. For example, massive sequencing allowed researchers to generate a complete genome of a methanogenic archaeon from the enrichment of a rice soil sample (Erkel et al., 2006). Similarly, Garcia Martín et al. obtained the draft genome of Candidatus *Accumulibacter phosphatis* strain UW-1 in the enhanced biological phosphorus removal (EBPR) active sluge sample (Garcia Martin et al., 2006). However, rare species in these samples can hardly get their complete genomes, and generally the assembled genome only represents a pan-genome owing to the presence of sub-species or horizontal gene transfer events. Spiking experiments of metagenomes with a pure culture isolate have suggested that a genome with little intra-species variation can be retrieved from a metagenome when it is covered at least 20 folds (Brown, 2015). There are several specific metagenome assemblers such as Meta-IDBA (Peng et al., 2011), MetaVelvet (Namiki et al., 2012), metaSPAdes (Nurk et al., 2017) and Ray Meta (Boisvert et al., 2012). Meta-Velvet and Meta-IDBA can distinguish reads from different species by partitioning the de Brujin graph based on k-mer coverage and separately assemble each sub-graph. Ray Meta does not decompose the de Brujin graph, but instead it uses a heuristics-guided graph traversal approach to find the optimal assembly. The outputs from various assemblers can be used to generate scaffolds using Bambus2 (Koren et al., 2011) to avoid miss-joins between distantly related organisms by detecting repeats and genomic variants.

Assembly of single-cell sequenced genome can avoid these difficulties caused by metagenomic sequencing. However, uneven sequence coverage, contaminated DNA and chimeric reads bring new assembly challenges. Besides those strategies mentioned above, there are several specific softwares for single-cell genome assembly such as Velvet-SC (Chitsaz et al., 2011), EULER+Velvet-SC (Chitsaz et al., 2011), IDBA-UD (Peng et al., 2012) and SPAdes (Bankevich et al., 2012). All of them are based on the de Bruijn graph and adapted for uneven read coverage. Velvet-SC optimizes the popular open source assembly program Velvet (Zerbino and Birney, 2008) by incorporating lower coverage sequences that are discard by most existing assemblers. Instead of filtering low coverage contigs, Velvet-SC merges them into a larger contig and recomputes their average coverage. E+V-SC is a software coupled with Velvet-SC with the error correction program EULER (Chaisson and Pevzner, 2008), which exhibits better performance on single-cell genome assembly. IDBA-UD uses multiple depth thresholds to remove erroneous k-mers in both low-depth and high-depth regions and an error-correction step is conducted to correct reads in high-depth regions to speed up the assembly process. SPAdes makes improvements based on E+V-SC, which can not only deal with non-uniform coverage but also remove chimeras. In addition, SPAdes further avoids making assembly decision solely based on coverage, but it can preserve low coverage regions that are discarded by other assemblers.

Although the single-cell genome assemblers mentioned above can perform metagenome assembly as well, recent studies demonstrated that the combined assembly of metagenome and single-cell genome can greatly improve the assembly continuity and completeness. For example, single-cell DNA extraction may cause chromosomal breaks or DNA damage that lead to the loss of some genomic regions (Rodrigue et al., 2009), which can be recovered from metagenomic data. On the other hand, reads from single-cell genome can provide clues for metagenome assembly. For example, several independent studies combined single-cell genomics and metagenomics to generate much improved bacterial genome assemblies from various bacterial communities (Dupont et al., 2012; Blainey, 2013; Nobu et al., 2015). Recently, Becraft et al. leveraged an existing single-cell genomic dataset from a candidate phylum Calescamantes (EM19) as anchors to calibrate a multi-layer perceptron machine learning algorithm and then generated metagenomic bins directly from sequencing reads of other samples (Becraft et al., 2015). In comparison to assembly-based methods, taxonomic binning with the read-based machine learning approach yielded final assemblies with much improved genome completeness. Ji et al employed flow cytometry to obtain a sorted mini-metagenome of the original sample and efficiently recovered high-quality genomes from the sorted mini-metagenome by the complementary of the original metagenome (Ji et al., 2017), which greatly improves the quality and quantity of novel microbial genomes. Alternatively, Yu et al used microfluidic parallelization to separate an environmental sample into many sub-samples containing 5–10 cells, and then they used co-occurrence information of genomes in each sub-sample to improve metagenome assembly (Yu et al., 2017). Collectively, the combination of metagenomics and single-cell genomics represents a promising direction for the assembly of uncultured microorganisms in the environment.

## Taxonomic clustering of contigs from metagenomic assemblies

A major challenge of obtaining complete genomes in metagenomic studies is to classify or bin the contigs from metagenomic assemblies into species- or strain-level clusters. Normally, there are two strategies, taxonomy-dependent classification (supervised) and taxonomy-independent classification (unsupervised). Taxonomy-dependent methods are based on sequence alignments, phylogenetic models and/or oligonucleotide patterns. Taxonomy-independent methods, however, extract features from contigs to infer bins based on sequence composition, abundance, marker genes, time series abundance profiles or any combination of them (Wu et al., 2014; Kang et al., 2015; Lin and Liao, 2016). However, these unsupervised binning methods do not perform well on samples with low-abundance species. MetaCluster 5.0 resolved this problem by separating high-abundance species reads from low-abundance species reads and using a two-round binning method (Wang et al., 2012). Due to the lack of reference genomes for metagenomic supervised classification, unsupervised approaches are the major strategy for metagenome binning. Recently, single-cell genomics is becoming an important anchor for supervised classification (Becraft et al., 2015), which can significantly improve the completeness of final assemblies compared with traditional binning approaches.

## Taxonomic and functional annotation of microbial genomes

SSU rRNA gene is widely used to determine the phylogenetic position of certain bacteria from environmental samples (Wu et al., 2009; Zaneveld et al., 2010). However, the lateral gene transfer may sometimes blur the result occasionally (Ochman et al., 2000). For example, it has been found that evolutionarily distant SSU rRNA genes were placed close together in phylogenetic trees (Woese et al., 1991; Hasegawa and Hashimoto, 1993). Therefore, inferring the phylogeny of microbes from the single SSU rRNA gene should be corroborated by the use of other phylogenetic markers such as functional genes. The concatenation of multiple universally distributed single copy genes performs better in phylogeny classification than the single SSU rRNA gene (Szollosi et al., 2012) as the combination of these phylogenetic signals will be more resistant to stochastic errors than those built from a single gene.

The last but not the least thing for single-cell metagenomics is to identify protein-coding genes, annotate their functions and reconstruct their metabolic pathways, through which we can know their physiological and metabolic characteristics. GLIMMER (Delcher et al., 1999; Delcher et al., 2007) is a widely used tool to identify coding genes from complete bacterial genomes. The annotation step is based on the homology search of genes against public databases such as KEGG, COG, EggNOG, NR etc. Most recently, several integrated pipelines (Overbeek et al., 2014; Seemann, 2014; Page et al., 2015) were developed, which provide high efficient and one-stop softwares for bacterial genome annotation.

## Application of single-cell metagenomics to virus-host interaction research

Virus-host interaction is a common process in environment, including infection, symbiosis and predation that can dynamically alter the evolution, diversity and metabolic potential of its host and finally affect the function of this interaction (De Smet et al., 2017). Traditionally, the studies of virus-host interaction were mainly based on laboratory experiments with pure cultures or indirect analyses of signatures using metagenomic approaches (Wang et al., 2016). However, the unculturable characteristic of most microbes limits such analysis of virus-host interaction, and similarly, metagenomics does not allow for the unambiguous identification of individual virus-host pairs. Single-cell genomics, owing to its culture-independent feature, can recover bacterial nuclear sequences and extra-chromosomal genetic elements in a cell simultaneously and thus can greatly facilitate cultivation-independent and cell-specific virus-host interaction studies. By using this approach, Yoon et al found a novel nanovirus from uncultivated Picozoan protist cells and obtained its whole genome sequence (Yoon et al., 2011). In another study, by analyzing 127 single amplified genomes (SAGs) of the uncultured gamma-proteobacterial clade SUP05, Roux et al found that a third of these cells were infected by Caudovirales (dsDNA) or Microviridae (ssDNA) bacteriophages (Roux et al., 2014). Similarly, Labonté et al. employed single-cell sequencing to analyze individual bacterial and archaeal cells simultaneously with viruses being inside or attached to them in their native environment (Labonte et al., 2015). They found that the viruses could be identified in 20 out of 58 phylogenetically and geographically diverse single amplified genomes (SAGs) of marine bacteria and archaea and at least four phage-host interactions had the characteristics of late lytic infections. This study demonstrates that single-cell genomics, in conjunction with sequence-based computational tools, enables in situ and cultivation-independent insights into host-virus interactions in complex microbial communities. Martinez-Garcia et al. combined SAG and microarrays to pinpoint the interaction between viruses and the ubiquitous hyperhalophilic Nanohaloarchaeota (Martinez-Garcia et al., 2014). Avital et al. used the scDual-Seq technology to analyze the interaction between intracellular pathogen *Salmonella typhimurium* and mouse macrophages, the RNA of the single-cell host macrophages cell and it’s infecting bacteria were sequenced simultaneously (Avital et al., 2017). They found three subpopulations of infected macrophages and many evidences of linear progression through these subpopulations which supporting a model that these three states correspond to consecutive stages of infection. Munson-McGee et al. combined single-cell sequencing with environmental metagenomics to explore the virus-host interactions in a Yellowstone National Park hot spring microbial community (Munson-McGee et al., 2018). They found that a broad spectrum of virus types from specialists to generalists coexist in a relatively low-diversity community. More than 60% of cells contain at least one virus type and a majority of these cells contain two or more virus types. Another study applied single-virus genomics and viral metagenomics to study the viral community structure of the oral cavity in human salivary samples (de la Cruz Peña et al., 2018), in which salivary viruses could be classified into about 200 major viral clusters, corresponding to approximately genus-level grouping. These studies demonstrate the power of single-cell metagenomics to unveil the diversity and genetic information of uncultured viruses in various communities.

## Application to comparative research of microbial subspecies

The differences among subspecies are hardly to determine when using metagenomic approaches, although a few methods have been developed to dissect strain-level diversities from metagenomes (Albanese and Donati, 2017; Quince et al., 2017; Truong et al., 2017). Single-cell genomics can help us to explore such differences. Using PCR procedure to confirm the subspecies after cell sorting, Kashtan applied large-scale single-cell sequencing to study the globally abundant marine cyanobacterium *Prochlorococcus* (Kashtan et al., 2014). They found that these *Prochlocococcus* spp. were composed of hundreds of subpopulations with distinct “genomic backbones”, each backbone consisting of a different set of core gene alleles linked to a small distinctive set of flexible genes. These subpopulations were estimated to have diverged at least a few million years ago, suggesting their ancient and stable niche partitioning. Such a large set of coexisting subpopulations may be a general feature of free-living bacterial species with huge populations in highly mixed habitats (Kashtan et al., 2014).

## Conclusion

The combination of single-cell genomics and metagenomics or metatranscriptomics can greatly improve our understanding of virus-host interaction, subspecies diversity, and gene expression for uncultured bacteria. Moreover, they can expand our view of microbial and functional diversity on both spatial and temporal scales. We believe that for an extended period of time, the benefits of single-cell genomics combined with metagenomics cannot be replaced. With the development of new sequencing technologies (longer reads and higher throughput) and better cell sorting methods, single-cell metagenomics will undoubtedly become an increasingly important approach for microbiome studies.

## Abbreviations

EBPR: enhanced biological phosphorus removal
epicPCR: emulsion, paired isolation and concatenation PCR
MDA: multiple displacement amplification
